# (2*Z*)-2-[(2,3-Dimethyl­phen­yl)imino]-1,2-diphenyl­ethanone

**DOI:** 10.1107/S1600536810028217

**Published:** 2010-07-21

**Authors:** Muhammad Ilyas Tariq, Muhammad Sarfraz, M. Nawaz Tahir, Shahbaz Ahmad, Ishtiaq Hussain

**Affiliations:** a Department of Chemistry, University of Sargodha, Sargodha, Pakistan; b Department of Physics, University of Sargodha, Sargodha, Pakistan

## Abstract

In the title compound, C_22_H_19_NO, the 2,3-dimethyl­anilinic group is planar with an r.m.s. deviation of 0.0226 Å. The phenyl rings with the carbonyl and imine substituents are also planar with r.m.s. deviations of 0.0019 and 0.0048 Å, respectively. These phenyl rings are oriented at dihedral angles of 74.70 (5) and 79.43 (5)°, respectively, with the 2,3-dimethyl­anilinic group, whereas the dihedral angle between them is 88.28 (4)°. Weak intra­molecular C—H⋯N hydrogen bonding occurs and completes an *S*(5) ring motif in the mol­ecule. In the crystal, weak π–π inter­actions are present between the carbonyl-containing phenyl rings at a centroid–centroid distance of 3.5958 (12) Å. C—H⋯π inter­actions between the 2,3-dimethyl­anilinic and the carbonyl-containing phenyl rings are also present, where the C—H group is from the former.

## Related literature

For title compound has been characterized as part of our programme for the synthesis of Schiff bases derived from 2,3-dimethylaniline, see: Hussain *et al.* (2010 ▶); Sarfraz *et al.* (2010 ▶); Tahir *et al.* (2010*a* ▶,*b* ▶); Tariq *et al.* (2010 ▶). For hydrogen-bond motifs, see: Bernstein *et al.* (1995 ▶).
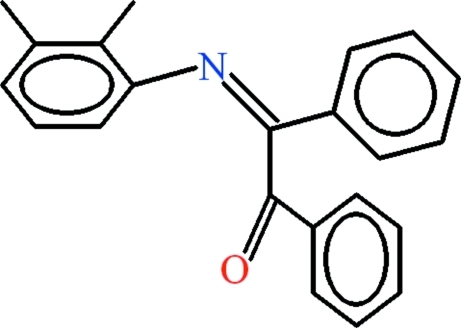


## Experimental

### 

#### Crystal data

C_22_H_19_NO
*M* *_r_* = 313.38
Monoclinic, 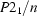

*a* = 13.3342 (3) Å
*b* = 8.7021 (2) Å
*c* = 15.6944 (5) Å
β = 108.448 (1)°
*V* = 1727.52 (8) Å^3^
*Z* = 4
Mo *K*α radiation
μ = 0.07 mm^−1^
*T* = 296 K
0.32 × 0.25 × 0.14 mm

#### Data collection

Bruker Kappa APEXII CCD diffractometer
Absorption correction: multi-scan (*SADABS*; Bruker, 2005 ▶) *T*_min_ = 0.982, *T*_max_ = 0.988
13196 measured reflections
3116 independent reflections
2296 reflections with *I* > 2σ(*I*)
*R*_int_ = 0.027

#### Refinement

*R*[*F* ^2^ > 2σ(*F* ^2^)] = 0.040
*wR*(*F* ^2^) = 0.119
*S* = 1.02
3116 reflections
219 parameters
H-atom parameters constrained
Δρ_max_ = 0.13 e Å^−3^
Δρ_min_ = −0.13 e Å^−3^

### 

Data collection: *APEX2* (Bruker, 2009 ▶); cell refinement: *SAINT* (Bruker, 2009 ▶); data reduction: *SAINT*; program(s) used to solve structure: *SHELXS97* (Sheldrick, 2008 ▶); program(s) used to refine structure: *SHELXL97* (Sheldrick, 2008 ▶); molecular graphics: *ORTEP-3 for Windows* (Farrugia, 1997 ▶) and *PLATON* (Spek, 2009 ▶); software used to prepare material for publication: *WinGX* (Farrugia, 1999 ▶) and *PLATON*.

## Supplementary Material

Crystal structure: contains datablocks global, I. DOI: 10.1107/S1600536810028217/bq2227sup1.cif

Structure factors: contains datablocks I. DOI: 10.1107/S1600536810028217/bq2227Isup2.hkl

Additional supplementary materials:  crystallographic information; 3D view; checkCIF report

## Figures and Tables

**Table 1:** Hydrogen-bond geometry (Å, °) *Cg*1 is the centroid of the C18–C23 ring.

*D*—H⋯*A*	*D*—H	H⋯*A*	*D*⋯*A*	*D*—H⋯*A*
C7—H7*B*⋯N1	0.96	2.38	2.849 (2)	109
C5—H5⋯*Cg*1^i^	0.93	2.99	3.6636 (19)	130

Symmetry code: (i) 
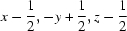
.

**Fig. 1.**
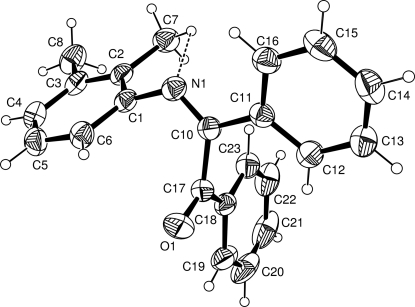
View of the title compound with the atom numbering scheme. The displacement ellipsoids are drawn at the 30% probability level. The dotted line represents the intramolecular H-bonding.

## supplementary crystallographic information

### Comment

The title compound (I, Fig. 1) is being reported in continuation to synthesize various Schiff bases (Hussain *et al.*, 2010; (Sarfraz *et al.*, 2010; Tahir *et al.*, 2010*a*; Tahir *et al.*, 2010*b*; Tariq *et al.*, 2010) of 2,3-dimethylaniline.

The crystal structures of (II) i.e, 2,3-dimethyl-*N*-[(*E*)-4-nitrobenzylidene]aniline (Tariq *et al.*, 2010), (III) *N*-[(*E*)-4-chlorobenzylidene]-2,3-dimethylaniline (Tahir *et al.*, 2010*a*), (IV) (*E*)-2,3-dimethyl-*N*-(2-nitrobenzylidene)aniline (Tahir *et al.*, 2010*b*), (V) 2,3-dimethyl-*N*-[(*E*)-2,4,5-trimethoxybenzylidene]aniline (Hussain *et al.*, 2010) and (VI) *N*-{(*E*)-[4-(dimethylamino)phenyl]methylidene}-2,3-dimethylaniline (Sarfraz *et al.*, 2010) have been published previously, which contain 2,3-dimethylaniline moiety. The title compound differs from these due to substitutions at the N-atom of 2,3-dimethylaniline.

In (I), the 2,3-dimethylanilinic group A (C1—C8/N1), the phenyl rings B (C11—C16) and C (C18—C23) are planar with r. m. s. deviation of 0.0226 Å, 0.0048 Å and 0.0019 Å, respectively. The dihedral angle between A/B, A/C and B/C is 79.43 (5)°, 74.70 (5)° and 88.28 (4)°, respectively. The central group D (C10/C17/O2) is oriented at 87.95 (9) ° and 5.37 (21)° with phenyl rings B and C, respectively. The title compound essentially consists of monomers. Weak intramolecular H-bonding of C—H···N type (Table 1, Fig. 1) exists and complete an S(5) ring motif (Bernstein *et al.*, 1995). There exists π–π interaction between the centroids of phenyl rings C at a distance of 3.5958 (12) Å [symmetry code: 1 - *x*, 1 - *y*, - *z*]. The C—H···π interaction (Table 1) also play an important role in stabilizing the molecules.

### Experimental

Equimolar quantities of 2,3-dimethylaniline and benzil were refluxed in methanol for 1 h. The yellow solution obtained was kept at room temperature to afford yellow prisms in 12 h.

### Refinement

All H-atoms were positioned geometrically (C–H = 0.93, 0.96 Å) and refined as riding with *U*_iso_(H) = *xU*_eq_(C), where *x* = 1.2 for aryl and *x* = 1.5 for methyl H-atoms.

### Crystal data

**Table:**

C_22_H_19_NO	*F*(000) = 664
*M**_r_* = 313.38	*D*_x_ = 1.205 Mg m^−^^3^
Monoclinic, *P*2_1_/*n*	Mo *K*α radiation, λ = 0.71073 Å
Hall symbol: -P 2yn	Cell parameters from 2296 reflections
*a* = 13.3342 (3) Å	θ = 1.8–25.3°
*b* = 8.7021 (2) Å	µ = 0.07 mm^−^^1^
*c* = 15.6944 (5) Å	*T* = 296 K
β = 108.448 (1)°	Prism, yellow
*V* = 1727.52 (8) Å^3^	0.32 × 0.25 × 0.14 mm
*Z* = 4	

### Data collection

**Table:**

Bruker Kappa APEXII CCD diffractometer	3116 independent reflections
Radiation source: fine-focus sealed tube	2296 reflections with *I* > 2σ(*I*)
graphite	*R*_int_ = 0.027
Detector resolution: 8.20 pixels mm^-1^	θ_max_ = 25.3°, θ_min_ = 1.8°
ω scans	*h* = −11→16
Absorption correction: multi-scan (*SADABS*; Bruker, 2005)	*k* = −10→10
*T*_min_ = 0.982, *T*_max_ = 0.988	*l* = −18→18
13196 measured reflections	

### Refinement

**Table:**

Refinement on *F*^2^	Primary atom site location: structure-invariant direct methods
Least-squares matrix: full	Secondary atom site location: difference Fourier map
*R*[*F*^2^ > 2σ(*F*^2^)] = 0.040	Hydrogen site location: inferred from neighbouring sites
*wR*(*F*^2^) = 0.119	H-atom parameters constrained
*S* = 1.02	*w* = 1/[σ^2^(*F*_o_^2^) + (0.0557*P*)^2^ + 0.2716*P*] where *P* = (*F*_o_^2^ + 2*F*_c_^2^)/3
3116 reflections	(Δ/σ)_max_ < 0.001
219 parameters	Δρ_max_ = 0.13 e Å^−^^3^
0 restraints	Δρ_min_ = −0.13 e Å^−^^3^

### Special details

**Table:**

Geometry. Bond distances, angles *etc*. have been calculated using the rounded fractional coordinates. All su's are estimated from the variances of the (full) variance-covariance matrix. The cell e.s.d.'s are taken into account in the estimation of distances, angles and torsion angles
Refinement. Refinement of *F*^2^ against ALL reflections. The weighted *R*-factor *wR* and goodness of fit *S* are based on *F*^2^, conventional *R*-factors *R* are based on *F*, with *F* set to zero for negative *F*^2^. The threshold expression of *F*^2^ > σ(*F*^2^) is used only for calculating *R*-factors(gt) *etc*. and is not relevant to the choice of reflections for refinement. *R*-factors based on *F*^2^ are statistically about twice as large as those based on *F*, and *R*- factors based on ALL data will be even larger.

### Fractional atomic coordinates and isotropic or equivalent isotropic displacement parameters (Å^2^)

**Table:**

	*x*	*y*	*z*	*U*_iso_*/*U*_eq_	
O1	0.33949 (10)	0.06238 (15)	−0.06560 (8)	0.0853 (5)	
N1	0.15316 (9)	0.23294 (14)	−0.01278 (8)	0.0570 (4)	
C1	0.14965 (10)	0.34191 (17)	−0.08138 (10)	0.0534 (5)	
C2	0.13588 (10)	0.49776 (18)	−0.06490 (10)	0.0557 (5)	
C3	0.12551 (11)	0.60342 (18)	−0.13434 (11)	0.0621 (5)	
C4	0.12628 (13)	0.5525 (2)	−0.21774 (11)	0.0691 (6)	
C5	0.13790 (14)	0.3994 (2)	−0.23354 (11)	0.0708 (6)	
C6	0.15002 (12)	0.29351 (19)	−0.16559 (10)	0.0632 (5)	
C7	0.13588 (14)	0.5497 (2)	0.02641 (11)	0.0749 (7)	
C8	0.11407 (16)	0.7732 (2)	−0.11967 (14)	0.0891 (7)	
C10	0.23292 (11)	0.14528 (16)	0.02009 (9)	0.0501 (5)	
C11	0.23229 (11)	0.02975 (17)	0.08880 (9)	0.0530 (5)	
C12	0.32515 (13)	−0.03892 (19)	0.14156 (11)	0.0663 (6)	
C13	0.32375 (16)	−0.1451 (2)	0.20646 (12)	0.0810 (7)	
C14	0.23045 (18)	−0.1860 (2)	0.21891 (13)	0.0847 (8)	
C15	0.13777 (16)	−0.1198 (2)	0.16724 (12)	0.0811 (7)	
C16	0.13822 (13)	−0.0116 (2)	0.10313 (11)	0.0656 (6)	
C17	0.33233 (11)	0.15133 (18)	−0.00799 (10)	0.0558 (5)	
C18	0.41638 (11)	0.25999 (18)	0.03844 (10)	0.0575 (5)	
C19	0.51182 (13)	0.2572 (2)	0.01995 (14)	0.0829 (7)	
C20	0.59158 (15)	0.3559 (3)	0.06489 (19)	0.1088 (10)	
C21	0.57832 (18)	0.4566 (3)	0.1268 (2)	0.1134 (10)	
C22	0.48486 (16)	0.4608 (2)	0.14604 (14)	0.0894 (8)	
C23	0.40401 (12)	0.36191 (19)	0.10183 (11)	0.0652 (6)	
H4	0.11880	0.62314	−0.26379	0.0830*	
H5	0.13760	0.36700	−0.29009	0.0849*	
H6	0.15842	0.18989	−0.17616	0.0758*	
H7A	0.20171	0.59963	0.05676	0.1123*	
H7B	0.12724	0.46230	0.06075	0.1123*	
H7C	0.07868	0.62036	0.02005	0.1123*	
H8A	0.11330	0.82866	−0.17276	0.1336*	
H8B	0.17250	0.80753	−0.06987	0.1336*	
H8C	0.04916	0.79136	−0.10707	0.1336*	
H12	0.38899	−0.01309	0.13306	0.0796*	
H13	0.38668	−0.18905	0.24194	0.0972*	
H14	0.22976	−0.25844	0.26227	0.1016*	
H15	0.07422	−0.14803	0.17551	0.0974*	
H16	0.07512	0.03397	0.06924	0.0787*	
H19	0.52156	0.18919	−0.02245	0.0995*	
H20	0.65549	0.35378	0.05280	0.1303*	
H21	0.63292	0.52304	0.15624	0.1360*	
H22	0.47601	0.52948	0.18847	0.1072*	
H23	0.34068	0.36404	0.11489	0.0783*	

### Atomic displacement parameters (Å^2^)

**Table:**

	*U*^11^	*U*^22^	*U*^33^	*U*^12^	*U*^13^	*U*^23^
O1	0.0909 (9)	0.0965 (10)	0.0757 (8)	0.0177 (7)	0.0368 (7)	−0.0170 (7)
N1	0.0486 (7)	0.0597 (7)	0.0594 (8)	−0.0032 (6)	0.0124 (6)	−0.0042 (6)
C1	0.0406 (7)	0.0582 (9)	0.0551 (8)	−0.0017 (6)	0.0063 (6)	−0.0042 (7)
C2	0.0390 (7)	0.0623 (9)	0.0591 (9)	0.0028 (6)	0.0061 (6)	−0.0078 (7)
C3	0.0453 (8)	0.0602 (9)	0.0688 (10)	0.0036 (7)	0.0008 (7)	−0.0033 (8)
C4	0.0623 (10)	0.0705 (11)	0.0626 (10)	0.0005 (8)	0.0027 (8)	0.0070 (8)
C5	0.0731 (10)	0.0782 (12)	0.0524 (9)	−0.0019 (9)	0.0076 (8)	−0.0075 (8)
C6	0.0651 (10)	0.0595 (9)	0.0571 (9)	−0.0011 (7)	0.0082 (7)	−0.0111 (8)
C7	0.0703 (11)	0.0791 (12)	0.0745 (11)	0.0095 (9)	0.0219 (9)	−0.0162 (9)
C8	0.0879 (13)	0.0651 (11)	0.0983 (14)	0.0132 (10)	0.0067 (11)	−0.0013 (10)
C10	0.0482 (8)	0.0526 (8)	0.0472 (8)	−0.0039 (6)	0.0120 (6)	−0.0107 (6)
C11	0.0563 (8)	0.0545 (8)	0.0489 (8)	−0.0050 (7)	0.0176 (7)	−0.0105 (6)
C12	0.0619 (10)	0.0688 (10)	0.0680 (10)	0.0039 (8)	0.0201 (8)	0.0054 (8)
C13	0.0876 (13)	0.0823 (12)	0.0716 (11)	0.0145 (10)	0.0230 (10)	0.0160 (10)
C14	0.1126 (16)	0.0796 (12)	0.0691 (12)	−0.0015 (12)	0.0389 (11)	0.0111 (10)
C15	0.0891 (13)	0.0915 (13)	0.0752 (12)	−0.0198 (11)	0.0436 (10)	−0.0038 (10)
C16	0.0601 (9)	0.0762 (11)	0.0629 (10)	−0.0075 (8)	0.0230 (8)	−0.0069 (8)
C17	0.0573 (9)	0.0609 (9)	0.0513 (8)	0.0110 (7)	0.0202 (7)	0.0044 (7)
C18	0.0468 (8)	0.0661 (9)	0.0608 (9)	0.0062 (7)	0.0188 (7)	0.0215 (8)
C19	0.0549 (10)	0.0988 (14)	0.1014 (14)	0.0199 (10)	0.0338 (10)	0.0461 (12)
C20	0.0447 (10)	0.131 (2)	0.146 (2)	0.0058 (13)	0.0237 (13)	0.0791 (18)
C21	0.0634 (14)	0.1087 (19)	0.136 (2)	−0.0285 (12)	−0.0142 (13)	0.0608 (17)
C22	0.0806 (13)	0.0796 (13)	0.0869 (14)	−0.0236 (10)	−0.0033 (10)	0.0119 (10)
C23	0.0568 (9)	0.0702 (10)	0.0652 (10)	−0.0108 (8)	0.0143 (8)	0.0057 (8)

### Geometric parameters (Å, °)

**Table:**

O1—C17	1.217 (2)	C20—C21	1.361 (4)
N1—C1	1.4243 (19)	C21—C22	1.373 (3)
N1—C10	1.2775 (19)	C22—C23	1.382 (3)
C1—C2	1.404 (2)	C4—H4	0.9300
C1—C6	1.389 (2)	C5—H5	0.9300
C2—C3	1.399 (2)	C6—H6	0.9300
C2—C7	1.503 (2)	C7—H7A	0.9600
C3—C4	1.385 (2)	C7—H7B	0.9600
C3—C8	1.510 (2)	C7—H7C	0.9600
C4—C5	1.373 (2)	C8—H8A	0.9600
C5—C6	1.380 (2)	C8—H8B	0.9600
C10—C11	1.476 (2)	C8—H8C	0.9600
C10—C17	1.524 (2)	C12—H12	0.9300
C11—C12	1.388 (2)	C13—H13	0.9300
C11—C16	1.390 (2)	C14—H14	0.9300
C12—C13	1.380 (2)	C15—H15	0.9300
C13—C14	1.366 (3)	C16—H16	0.9300
C14—C15	1.372 (3)	C19—H19	0.9300
C15—C16	1.379 (2)	C20—H20	0.9300
C17—C18	1.471 (2)	C21—H21	0.9300
C18—C19	1.392 (2)	C22—H22	0.9300
C18—C23	1.381 (2)	C23—H23	0.9300
C19—C20	1.375 (3)		
			
O1···N1	3.2197 (19)	C11···H14^iv^	2.8900
O1···C6	3.222 (2)	C11···H8B^v^	3.0500
O1···C19^i^	3.359 (2)	C12···H14^iv^	3.0800
O1···H6	2.7200	C14···H7A^v^	3.0800
O1···H19	2.5600	C14···H23^vi^	3.0800
O1···H19^i^	2.9200	C17···H6	2.9300
N1···O1	3.2197 (19)	C17···H12	2.5400
N1···C23	3.446 (2)	C18···H12	2.8900
N1···H7B	2.3800	C20···H5^vii^	2.9000
N1···H16	2.5700	C21···H5^vii^	3.1000
N1···H23	2.9000	H4···H8A	2.3000
C1···C18	3.530 (2)	H5···C20^viii^	2.9000
C3···C20^ii^	3.598 (3)	H5···C21^viii^	3.1000
C6···C17	3.123 (2)	H6···O1	2.7200
C6···O1	3.222 (2)	H6···C10	2.9500
C7···C7^iii^	3.559 (3)	H6···C17	2.9300
C12···C18	3.480 (2)	H7A···C8	3.0400
C17···C6	3.123 (2)	H7A···C14^ix^	3.0800
C18···C12	3.480 (2)	H7B···N1	2.3800
C18···C1	3.530 (2)	H7C···C8	2.7300
C18···C21^ii^	3.596 (3)	H7C···H8C	2.4200
C19···C21^ii^	3.347 (3)	H7C···C7^iii^	3.1000
C19···C22^ii^	3.589 (3)	H8A···H4	2.3000
C19···O1^i^	3.359 (2)	H8B···C7	2.8300
C20···C3^ii^	3.598 (3)	H8B···C11^ix^	3.0500
C20···C21^ii^	3.540 (4)	H8C···C7	2.9400
C20···C22^ii^	3.522 (3)	H8C···H7C	2.4200
C21···C19^ii^	3.347 (3)	H8C···H16^iii^	2.4600
C21···C20^ii^	3.540 (4)	H12···C17	2.5400
C21···C18^ii^	3.596 (3)	H12···C18	2.8900
C22···C19^ii^	3.589 (3)	H14···C11^vi^	2.8900
C22···C20^ii^	3.522 (3)	H14···C12^vi^	3.0800
C23···N1	3.446 (2)	H16···N1	2.5700
C2···H20^ii^	3.0200	H16···H8C^iii^	2.4600
C3···H20^ii^	2.8200	H16···H16^x^	2.5200
C5···H21^ii^	2.9900	H19···O1	2.5600
C7···H8B	2.8300	H19···O1^i^	2.9200
C7···H8C	2.9400	H20···C2^ii^	3.0200
C7···H7C^iii^	3.1000	H20···C3^ii^	2.8200
C7···H23	3.1000	H21···C5^ii^	2.9900
C8···H7A	3.0400	H23···N1	2.9000
C8···H7C	2.7300	H23···C7	3.1000
C10···H6	2.9500	H23···C10	2.5600
C10···H23	2.5600	H23···C14^iv^	3.0800
			
C1—N1—C10	121.79 (13)	C4—C5—H5	120.00
N1—C1—C2	118.62 (13)	C6—C5—H5	120.00
N1—C1—C6	120.55 (13)	C1—C6—H6	120.00
C2—C1—C6	120.57 (14)	C5—C6—H6	120.00
C1—C2—C3	118.56 (14)	C2—C7—H7A	109.00
C1—C2—C7	120.39 (14)	C2—C7—H7B	109.00
C3—C2—C7	121.03 (14)	C2—C7—H7C	109.00
C2—C3—C4	119.84 (15)	H7A—C7—H7B	109.00
C2—C3—C8	120.93 (15)	H7A—C7—H7C	109.00
C4—C3—C8	119.23 (15)	H7B—C7—H7C	109.00
C3—C4—C5	121.08 (15)	C3—C8—H8A	109.00
C4—C5—C6	120.06 (15)	C3—C8—H8B	109.00
C1—C6—C5	119.89 (15)	C3—C8—H8C	109.00
N1—C10—C11	120.34 (14)	H8A—C8—H8B	109.00
N1—C10—C17	123.48 (13)	H8A—C8—H8C	109.00
C11—C10—C17	116.18 (13)	H8B—C8—H8C	109.00
C10—C11—C12	121.24 (14)	C11—C12—H12	120.00
C10—C11—C16	120.57 (14)	C13—C12—H12	120.00
C12—C11—C16	118.19 (14)	C12—C13—H13	120.00
C11—C12—C13	120.71 (17)	C14—C13—H13	120.00
C12—C13—C14	120.38 (18)	C13—C14—H14	120.00
C13—C14—C15	119.79 (18)	C15—C14—H14	120.00
C14—C15—C16	120.5 (2)	C14—C15—H15	120.00
C11—C16—C15	120.48 (17)	C16—C15—H15	120.00
O1—C17—C10	118.21 (14)	C11—C16—H16	120.00
O1—C17—C18	123.39 (15)	C15—C16—H16	120.00
C10—C17—C18	118.35 (13)	C18—C19—H19	120.00
C17—C18—C19	119.27 (14)	C20—C19—H19	120.00
C17—C18—C23	121.58 (14)	C19—C20—H20	120.00
C19—C18—C23	119.13 (15)	C21—C20—H20	120.00
C18—C19—C20	119.46 (18)	C20—C21—H21	120.00
C19—C20—C21	120.9 (2)	C22—C21—H21	120.00
C20—C21—C22	120.4 (2)	C21—C22—H22	120.00
C21—C22—C23	119.4 (2)	C23—C22—H22	120.00
C18—C23—C22	120.62 (16)	C18—C23—H23	120.00
C3—C4—H4	119.00	C22—C23—H23	120.00
C5—C4—H4	119.00		
			
C10—N1—C1—C2	−121.60 (15)	N1—C10—C17—C18	86.93 (18)
C10—N1—C1—C6	64.3 (2)	C11—C10—C17—O1	84.26 (17)
C1—N1—C10—C11	−177.78 (13)	C11—C10—C17—C18	−93.34 (16)
C1—N1—C10—C17	2.0 (2)	C10—C11—C12—C13	179.05 (15)
N1—C1—C2—C3	−175.88 (13)	C16—C11—C12—C13	−0.1 (2)
N1—C1—C2—C7	6.1 (2)	C10—C11—C16—C15	179.87 (15)
C6—C1—C2—C3	−1.7 (2)	C12—C11—C16—C15	−1.0 (2)
C6—C1—C2—C7	−179.80 (15)	C11—C12—C13—C14	1.0 (3)
N1—C1—C6—C5	174.71 (15)	C12—C13—C14—C15	−0.7 (3)
C2—C1—C6—C5	0.7 (2)	C13—C14—C15—C16	−0.4 (3)
C1—C2—C3—C4	1.6 (2)	C14—C15—C16—C11	1.2 (3)
C1—C2—C3—C8	−177.99 (15)	O1—C17—C18—C19	−3.6 (2)
C7—C2—C3—C4	179.68 (16)	O1—C17—C18—C23	178.28 (16)
C7—C2—C3—C8	0.1 (2)	C10—C17—C18—C19	173.91 (15)
C2—C3—C4—C5	−0.5 (3)	C10—C17—C18—C23	−4.3 (2)
C8—C3—C4—C5	179.14 (18)	C17—C18—C19—C20	−178.35 (19)
C3—C4—C5—C6	−0.6 (3)	C23—C18—C19—C20	−0.1 (3)
C4—C5—C6—C1	0.5 (3)	C17—C18—C23—C22	178.62 (16)
N1—C10—C11—C12	−163.48 (14)	C19—C18—C23—C22	0.5 (3)
N1—C10—C11—C16	15.6 (2)	C18—C19—C20—C21	−0.3 (4)
C17—C10—C11—C12	16.8 (2)	C19—C20—C21—C22	0.4 (4)
C17—C10—C11—C16	−164.10 (14)	C20—C21—C22—C23	−0.1 (4)
N1—C10—C17—O1	−95.47 (19)	C21—C22—C23—C18	−0.3 (3)

Symmetry codes: (i) −*x*+1, −*y*, −*z*; (ii) −*x*+1, −*y*+1, −*z*; (iii) −*x*, −*y*+1, −*z*; (iv) −*x*+1/2, *y*+1/2, −*z*+1/2; (v) *x*, *y*−1, *z*; (vi) −*x*+1/2, *y*−1/2, −*z*+1/2; (vii) *x*+1/2, −*y*+1/2, *z*+1/2; (viii) *x*−1/2, −*y*+1/2, *z*−1/2; (ix) *x*, *y*+1, *z*; (x) −*x*, −*y*, −*z*.

### Hydrogen-bond geometry (Å, °)

**Table:**

Cg1 is the centroid of the C18–C23 ring.

**Table:**

*D*—H···*A*	*D*—H	H···*A*	*D*···*A*	*D*—H···*A*
C7—H7B···N1	0.96	2.38	2.849 (2)	109
C5—H5···Cg1^viii^	0.93	2.99	3.6636 (19)	130

Symmetry codes: (viii) *x*−1/2, −*y*+1/2, *z*−1/2.
